# New insight into the effects of lead modulation on antioxidant defense mechanism and trace element concentration in rat bone

**DOI:** 10.2478/v10102-009-0003-5

**Published:** 2009-03

**Authors:** Bhardwaj Payal, Harkiran Preet Kaur, Durg Vijay Rai

**Affiliations:** Department of Biophysics, Panjab University, Chandigarh, 160014, India

**Keywords:** bone, lead toxicity, DNA, RNA, ALP, antioxidant enzymes and trace element analysis

## Abstract

Risks of heavy metals-induced severe bone disorders generate interest to their toxicity. The present study was undertaken to monitor the biochemical and antioxidant status of bone of 30 and 80 days old male Wistar rats exposed to 5 week lead treatment. At the end of study, the rats were sacrificed, their long bone i.e. femur were excised, cleaned of soft tissue, minced and homogenized. Nucleic acid content, alkaline phosphatase, lipid peroxidation, catalase, glutathione S-transferase and superoxide dismutase were determined in bone. In both groups of treated animals lead treatment increased the production of malondialdehyde, while reducing activities of catalase, glutathione S-transferase and superoxide dismutase, indicating that it causes oxidative stress. Parallely with these effects lead significantly reduced the nucleic acid content and the activity of alkaline phosphatase, considered as biomarkers of osteoblast's function, conditions and development of bones. Moreover the concentrations of copper, zinc, iron and sodium were reduced in the excised bones. The present study indicates that the lead induced bone toxicity and its deteriorated development is the consequence of a primary oxidative stress. Our results may be helpful in understanding the modulation of biochemical parameters under lead toxicity.

## Introduction

Lead is one of the first discovered and widely known metals commonly encountered in the environment (Shoty *et al*., 1998). Lead poisoning is one of the foremost environmental health threats (Juberg *et al*., 1997). Humans lead poisoning develops if the people are exposed to harmful levels of lead-contaminated dust from its continued release into the environment as an exhaust emission product of leaded gasoline, as well as due to its wide spread industrial use (deteriorated lead-based paints and lead-contaminated soil). Immediately after absorption, lead enters into the blood, which is then distributed particularly to the liver and kidneys, and is then stored in the bones and cause damage of liver, kidneys, heart, male gonads and affects immune and nervous system. Millions of children have enough lead in their blood to reduce intelligence and attention span, cause learning disabilities, and damage permanently a child's brain and nervous system (ATSDR 2005, 2007).

Metal-induced toxicity, with an emphasis on the generation and role of reactive oxygen species (ROS) in case of bone is not well known. Metal-mediated formation of free radicals causes various modifications to deoxyribonucleic acid (DNA) bases, enhances lipid peroxidation, and alters calcium and sulfhydryl homeostasis. Lipid peroxides, formed by the attack of radicals on polyunsaturated fatty acid residues of phospholipids, can further react with redox metals finally producing mutagenic and carcinogenic malondialdehyde (MDA), 4-hydroxynonenal and other exocyclic DNA adducts (etheno and/or propano adducts).

The primary route for heavy metal toxicity is depletion of glutathione and bonding to sulfhydryl groups of proteins. Lead toxicity damages cellular material and alters cellular genetics (Lyn, 2006). The pathogenesis of lead toxicity is multifactorial, as lead causes oxidative stress by inducing the generation of ROS, reducing the antioxidant defense system of cells via depleting glutathione, interfering with some essential metals, inhibiting sulfhydryl dependant enzymes or antioxidant enzymes activities and/or increasing susceptibility of cells to oxidative attack by altering membrane integrity and fatty acid composition (Inouye *et al*., 2001; Hande and Naran, 2000). Antioxidants are the compounds that protect cells against ROS or free radicals in the body. Under physiological conditions, ROS are cleared from the cells by the action of superoxide dismutase (SOD), catalase (CAT), or glutathione peroxidase (GPX). When free radicals or oxidants are produced in abundance, cells suffer from oxidative stress. Fortunately, the balance between free radical generation in tissues and endogenous antioxidants activity prevents oxidative stress in physiological conditions. Thus cellular health depends on maintenance of this balance. The free-radicals are named troublemakers and originate mostly from ROS in the mammalian tissues. Free-radicals acquire an electron from the molecule next to it, then that molecule or atom may become a free-radical. It becomes unstable and will try to take another electron from any other molecule in its immediate environment, and so on. Thus, there is a chain reaction of molecules that are desperately seeking completion, leaving severe damage in their surroundings wherever an electron pair is broken. If free radicals capture electrons from lipids of cell membranes, the evoked lipid peroxidation damages the membrane functions.

CAT, SOD, Glutathione S-transferases (GST) and GPX are the main endogenous antioxidant enzymes. CAT commonly found in living tissue consists of four subunits, containing heme group with iron in the active center. This enzyme converts toxic peroxides to harmless products of water and oxygen (Luck, 1971). The production of hydrogen peroxide (H_2_O_2_) in eukaryotic cells is an end product of the reaction between various oxidases and SOD. Accumulation of H_2_O_2_ may damage the cells due to oxidation of proteins, DNA, and lipids, thus causing cell death and mutagenesis (Tada-Oikawa *et al*., 1999; Kowaltowski *et al*., 2000). Recent studies brought evidence that oxidative stress modulates osteoblastic differentiation of bone cells (Mody *et al*., 2001). SOD is a protein with copper and zinc, or manganese as cofactors and catalyzes dismutation of superoxide (O_2_·^−^) into oxygen and H_2_O_2_ (Frodovich, 1995). GSTs catalyse the conjugation of reduced glutathione via the sulfhydryl group, to electrophilic centers on wide variety of substrates. This activity is useful in detoxification of products of oxidative stress, such as peroxidised lipids (Hayes and Pulford, 1995). GPX is an enzyme with peroxidase activity whose main biological role is to protect the organism from oxidative damage. The biochemical function of GPX is to reduce lipid hydroperoxides to their corresponding alcohols and to reduce free H_2_O_2_ to water (Muller *et al*., 2007).

Most research about lead exposure on various antioxidant enzymes and tissues has been mainly experimental and the results are often divergent. The purpose of our study was therefore to evaluate the activity of endogenous antioxidant enzymes and the production of ROS in bone with reference to other bone marker. Biochemical changes including nucleic acid content, alkaline phosphatase (ALP) activity, antioxidant defense system enzymes and malodialdehydes (MDA) level of femur under lead administration were monitored. The study paves the path in understanding the cellular basis of lead induced alterations leading to changes in bone metabolism.

## Methods

### Experimental modality

To carry out the present investigation, male Wistar rats were procured from the Central Animal House of Panjab University, Chandigarh. The animals were housed in polypropylene cages bedded with sterilized rice husk. They were given free access to clean drinking water (tap water) and standard animal pellet diet (Ashirwad Industries, Kharar, Punjab, India), throughout the experiment. The temperature of the animal room was maintained at 21±1°C, humidity 50–60% and dark and light cycle was 12 hrs. The animals were acclimatized for one week to laboratory conditions before the experiment. The experimental protocols were approved by the Institutional Ethics Committee and conducted according to Indian National Science Academy Guidelines for the use and care of experimental animals.

### Chemicals used

Lead acetate (~99% pure) was purchased from Qualigens Fine Chemicals: A division of Glaxo Smith Kline Pharmaceuticals Limited, Mumbai, India. Ethylene diamine tetraacetic acid (EDTA), Diaphenylamine (DPA) and Orcinol from S.D fine chemicals Ltd. Paranitro phenyl phosphate (PNPP), Hydrogen peroxide, Thio barbituric acid (TBA), Trichloroacetic acid and glacial acetic acid from E. Merk (India) Pvt. Ltd, Sisco research lab Pvt Ltd.

### Experimental design

**Figure 1:**
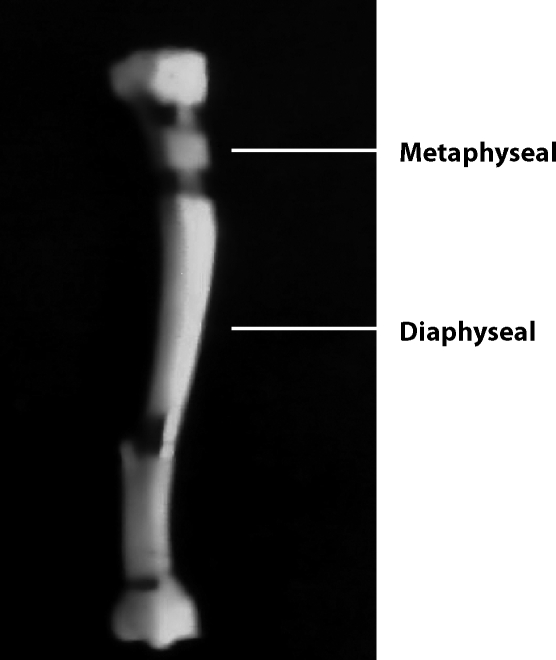
Division of femur to metaphyseal and diaphyseal part.

Forty male Wistar rats were taken in two age groups i.e. 30 day's old (controls /CI/ and experimental /Lead I/) and 80 day's old (controls /CII/ and experimental /Lead II/) animals. To produce a subclinical toxicity, lead (as lead acetate 250 mg/ml) was provided ad libitum in drinking water for five weeks. Glacial acetic acid was added to the drinking water of lead administered groups at a concentration of 12.5 μ/l to prevent the precipitation of lead acetate. Blood was collected by puncturing the ocular vein before scarification, kept at room temperature for 2–3 hrs and then centrifuged at 1290 × g for 15 min to obtain serum. The obtained serum was used for analysis of ALP. At the end of the study, rats from all four groups were sacrificed by decapitation and their long bone i.e. femurs were excised, cleaned of soft tissue. The femurs were cut into the metaphyseal and diaphyseal part (Figure 1) with the help of bone cutter and then these parts were minced and homogenized 10% of their total weight in sodium phosphate buffer (0.1 M, pH7.5) in pestle mortar and were centrifuged at 10,000 g for 30 min. The supernatant was collected and used for biochemical assays (SOD, CAT, GST and LPO).

### Estimation of nucleic acids

To measure nucleic acid content of respective bone powders, they were defatted with chloroform/methanol (1: 1 in volume) overnight. Then the residue was demineralized in 0.5 M EDTA/50 mM Tris-HCl, pH7.4 containing 1M NaCl. Demineralization was continued for two weeks and then the nucleic acid contents were estimated. The demineralized bone powder was dissolved in 0.1 ml of 0.1 N NaOH. 20μl of sample was diluted to 100μl by addition of 80μl of distilled water and used for estimation of DNA and RNA. The DNA and RNA were determined by the method of Plummer (Plummer, 1988). In case of DNA, to 2 ml of above sample (extract) 4 ml of diphenylamine (DPA) reagent was added and the samples were kept in boiling water bath at 90°C for 10 min. After cooling, the samples were read at 595 nm and DNA content was estimated using the linear standard curve of standard resolution of DNA.

In case of RNA, to 2 ml of sample (extract), 3 ml of Orcinol reagent was added and the samples were kept in boiling water bath at 90°C for 15 min. After completion of incubation period the samples were cooled and the color formed was read at 660 nm.

### Assessment of ALP

For ALP estimation, bone tissue was minced and homogenized in 3 ml of an ice-cold 6.5 mM barbital buffer (pH7.4) and ultracentrifuged for 60 s. Further, it was centrifuged for 5 min at 3000 rpm, and its supernatant fraction was used to measure ALP activity. The activity of ALP was estimated according to the method of Bergmeyer *et al*. (1980). To 900μl PNPP (Para nitro phenyl phosphate), 100μl distilled water and 1.3 ml glycine buffer were added and incubated for 5 min at 37°C. Then 200μl of enzyme extract was added and tubes were further incubated for 30–45 min at 37°C. To stop the reaction 3.5 ml of 0.1 N NaOH was added and liberated PNP (Para nitro phenol) was measured by spectrophotometer at 420 nm. The enzymatic activity was expressed as enzyme protein content (mg) using PNP content (nmol) produced by reaction through incubation for one minute.

### Assessment of CAT

CAT was estimated directly as described by Luck (1971).

### Assessment of SOD

The activity of SOD was estimated according to the method of Kono (1978).

### Assessment of GST

GST activity was determined spectrophotometrically according to the method of Habig *et al*. (1973).

### MDA assay

LPO in femur was performed according to the method of Devasagayam *et al*. (1987). The reaction mixture consisted of 0.1 ml 0.15 mol/l Tris-HCl buffer (pH7.4), 0.3 ml of 10mmol/l KH_2_PO_4_ and 0.2 ml of tissue extract in a total volume of 2 ml. The reaction mixture was incubated at 37°C for 20 min with constant shaking. The reaction was stopped by the addition of 1 ml of 10% TCA. The tubes were shaken well and 1.5 ml TBA was added and heated in a boiling water bath for 20 min. The pink colored complex was formed whose absorbance was read at 532 nm. The amount of MDA formed (index of lipid peroxidation) was calculated using an extinction coefficient of 1.56×10^5^ M^–1^cm^–1^ for MDA-TBA chromophore and the results are expressed as nmoles of MDA formed /min/mg of protein.

### Protein estimation

Protein was estimated by the method of Lowry *et al*. (1951).

### Elemental analysis (Atomic Absorption Spectroscopy)

Trace elemental analysis for bone samples was done using the Perkin Elmer, Intensitron Atomic Absorption Spectrometer (Model 3100). For atomic absorption, bones were digested in 0.5 M EDTA (demineralizing solution). The bones were allowed to demineralize for 96 hrs at room temperature. After demineralization the bones were dried and weight loss was found. The bones were considered to be fully demineralized if the weight loss was –70% of the dry weight. The demineralizing solution was used as a sample for elemental analysis.

### Statistical Analysis

Statistics analysis of the data was performed by analysis of variances (one way ANOVA). Following one way ANOVA post Hoc test using least significance difference (LSD) and by student ‘t’ test at p = 0.05 using SPSS statistical software data for individual parameters represents average value calculated from three parallels.

## Results

Significant decrease (*p*<0.001) in DNA and RNA content of femoral diaphyseal and metaphyseal tissue was observed in experimental groups as compared to their respective controls (Table 1). About 35.02% and 43.9% reduction was found in the metaphyseal DNA, whereas 48.3% and 49.1% reduction was found in the diaphysial DNA of both age groups, respectively. Similarly reduction of 35.86% and 53% was found in the metaphyseal RNA in both age groups.

**Table 1:** Nucleic acid content (DNA/RNA).

	DNA (µgm/mg wet tissue)		RNA (µgm/mg wet tissue)	
		
Groups	Metaphyseal tissue	Diaphyseal tissue	Metaphyseal tissue	Diaphyseal tissue
CI	3.34±0.13	1.78±0.04	2.37±0.23	1.50±0.05
Lead I	2.17±0.10^***^	0.92±0.01^***^	1.52±0.07^***^	0.80±0.05^***^
CII	3.57±0.22	1.85±0.06	3.00±0.12	1.67±0.07
Lead II	2.00±0.05^***^	0.94±0.03^**^	1.41±0.25^***^	0.82±0.05^***^

Data: Mean±Standard Deviation (N=10).

Statistically significant differences are represented by:

^*^ *p*≤0.05,

^**^ *p*≤0.01,

^***^ *p*≤0.001 lead treated group w.r.t. control

ALP activity significantly (*p*<0.001) decreased in both the diaphyseal and metaphyseal femoral tissue of treated groups, as compared to their respective controls. Similar trend was seen in ALP activity of serum in which there was a reduction in the activity to about 57.3% and 61.1% in the two age groups, respectively (Table 2).

**Table 2:** Alkaline Phosphatase (ALP) activity in bone and serum.

Groups	ALP Activity (µmoles/min/mg protein) in bone		Serum ALP Activity (10^−3^µmoles/min/mg protein)
	
	Metaphyseal tissue	Diaphyseal tissue	
CI	2.81±0.28	1.68±0.07	5.34±0.25
Lead I	1.33±0.07^***^	0.99±0.05^***^	2.28±0.04^***^
CII	3.33±0.14##	1.74±0.07	5.64±0.28#
Lead II	1.28±0.01^***^	0.94±0.10^**^	2.19±0.12^***^

Data: Mean±Standard Deviation (N=10).

Statistically significant differences are represented by:

^*^ *p*≤0.05,

^**^ *p*≤0.01,

^***^ *p*≤0.001 lead treated group w.r.t. control,

#*p*≤0.05,

##*p*≤0.01,

###*p*≤0.001 CII w.r.t CI

Lead treatment increased the level of MDA almost to the same extent, as compared to their respective control values in the young (by 47.7%) and old animals (47.1%) (Table 3). Lead treatment reduced the activity of the antioxidant enzymes CAT, SOD and GST in femur of lead treated groups both in young and old animals when compared to their respective controls (Table 3). Trace elemental analysis by atomic absorption spectroscopy revealed reduction in the concentration of Cu, Zn, Fe and Na ions when compared with their respective controls (Table 4). We have found reduction in the concentration of Cu to about 38.5%, Zn to about 30.45%, Fe to about 14.82% and Na to about 26.50% for both age groups.

**Table 3:** CAT, SOD, GST activity and LPO in bone.

Groups	LPO (nmoles MDA) formed/mg protein	Catalase (µmoles H_2_O_2_) formed/min/mg protein	SOD (Units/mg protein)	GST (nmoles of conjugate) formed/min/mg protein
CI	2.74±0.06	19.83±0.74	12.50±1.63	2.12±0.13
Lead I	5.74±0.008^***^	10.61±0.91^***^	8.99±0.71^**^	1.22±0.16^***^
CII	2.75±0.06	21.66±1.32##	14.25±0.92#	2.22±0.08#
Lead II	5.84±0.07^***^	12.42±1.27^***^	7.12±0.63^***^	1.10±0.04^***^

Data: Mean±Standard Deviation (N=10).

Statistically significant differences are represented by:

^*^ *p*≤0.05,

^**^ *p*≤0.01,

^***^ *p*≤0.001 lead treated group w.r.t. control,

#*p*≤0.05,

##*p*≤0.01,

###*p*≤0.001 CII w.r.t CI

**Table 4:** Trace elemental analysis.

	Concentration (ppm)			
	
Groups	Copper (Cu)	Zinc (Zn)	Iron (Fe)	Sodium (Na)
CI	1.27±0.15	1.74±0.04	6.34±0.21	1.66±0.09
Lead I	0.78±0.01^***^	1.21±0.07^***^	5.40±0.16^***^	1.22±0.05^***^
CII	1.32± 0.31	1.82±0.03	6.62±0.17	1.81±0.12
Lead II	0.76±0.02^**^	1.27±0.03^***^	5.19±0.11^***^	1.08±0.07^***^

Data: Mean±Standard Deviation (N=10).

Statistically significant differences are represented by:

^*^ *p*≤0.05,

^**^ *p*≤0.01,

^***^ *p*≤0.001 lead treated group w.r.t. control

## Discussion

In the present study, decrease in ALP activity and nucleic acid content was observed in both diaphyseal (cortical bone) and metaphyseal (trabecular bone) femoral tissue which infer that lead toxicity has effect on both cortical and trabacular bones. Effect of lead toxicity on bone cell function has been reported earlier (Pounds *et al*., 1991). DNA content in bone tissues is an index of the number of bone cells (Canalis *et al*., 1989). Bone ALP is the enzyme marker of osteoblast activity, and hence participates in the bone mineralization process (Majeska and Wurthier, 1975). Earlier reports have shown the inhibitory effect of lead on osteoblastic expression (Klein and Wiren, 1993). Through its action to inhibit nucleic acid content and osteoblastic activity, lead may limit attainment of peak bone mass and disrupt the tight coupling of bone formation and resorption necessary for normal bone modeling. Various reports suggested correlation between mRNA levels for bone cell proteins and bone formation in long bones of maturing rats (Turner and Spelsberg, 1991). ALP activity also decreases in serum. Earlier experiments have also shown that lead induced inhibition of specific steady state mRNA levels is responsible for reduction in ALP activity (Klein and Wiren, 1993). Our results clearly demonstrated that lead impairs the nucleic acids metabolism and ALP activity. Lead has more effect on developing skeleton or children as compared to adults because children have increased absorption rates and retention levels and lead burden (Shih Hu *et al*., 2007; Smith *et al*., 2008).

To fully understand the significance of bone as a target tissue of lead toxicity, as well as a reservoir of systemic lead, it is necessary to define the effects of lead on the cellular and enzymatic components of bone. So, in the present study, various antioxidant enzymes were estimated to scrutinize the effect of lead on antioxidant defense system of body. Lead induced oxidative stress, increased the levels of MDA in the process of LPO. MDA has got high reactivity towards amino groups; it inhibits the synthesis of nucleic acids and proteins and also deactivates the enzymes (Bird and Draper, 1980). Thus the decrease in femur antioxidant enzymes activities and nucleic acid levels observed after lead treatment may be due to heightened LPO. Lead treatment significantly decreased the activity of SOD, CAT and GST when compared to control groups. The decrease in SOD activity may result in accumulation of O_2_·^−^, which has been shown to inhibit other antioxidant enzymes (Kono and Fridovich, 1982). SOD is responsible for O_2_ ·^−^ dismutation to H_2_O_2_, a species that is longer active than O_2_ ·^−^. Lead toxicity leads to free radical damage via generation of ROS or by direct depletion of antioxidant reserves (Ercal *et al*., 2001). A significant decrease in CAT suggests an increased utilization of CAT in response to ROS in the process converting H_2_O_2_ to water. There is strong relationship between oxidative stress and bone loss (Sánchez-Rodríguez *et al*., 2007; Liu *et al*., 2008; Shen *et al*., 2008). Trace elements are required for proper functioning of various enzymes. SOD, an antioxidant defense enzyme requires copper and zinc as cofactors. Copper is required by lysyl oxidase that catalyses cross linking of lysine and hydroxyproline in collagen, contributing to the mechanical strength of collagen fibrils. ALP and collagenase also utilize zinc as cofactor. Since copper and zinc play an important role in bone metabolism and turn over decrease in their concentration as shown in the present study interfere with bone development and functioning. Significant decrease in the amount of trace elements in lead treated groups reflects the disruption in collagen synthesis, bone turnover and metabolism. The decrease in the trace element concentration can also be correlated with the decrease in various enzymes activities as these elements are recognized as the cofactors of various enzymes.

Based on our results we may assume, that increased oxidative stress in lead treated bone results from the accelerated formation of oxidants and reduced antioxidant defense capacity of the bone cells or by both. These results may pave a new pathway in understanding of mechanism of lead toxicity on bone.
